# Acute Poisoning Due to an Intentional Overdose of 2,4-Dichlorophenoxyacetic Acid

**DOI:** 10.7759/cureus.80116

**Published:** 2025-03-05

**Authors:** Gurnoor Dhaliwal, Karanveer S Brar, Jasprabh Kaur, Alina Ishaque, Jasleen Kaur

**Affiliations:** 1 Emergency Medicine, Max Superspeciality Hospital, Mohali, IND; 2 Internal Medicine, Ambedkar State Institute of Medical Sciences, Mohali, IND; 3 Internal Medicine, CarePoint Health Bayonne Medical Center, Bayonne, USA; 4 Endocrinology, Diabetes and Metabolism, HealthPartners, Minneapolis, USA

**Keywords:** dichlorophenoxyacetic acid poisoning, herbicide, intentional overdose, rhabdomyolysis, urine alkalinization

## Abstract

Ethyl ester, chemically known as 2,4-dichlorophenoxyacetic acid (2,4-D), is a widely-used herbicide. It is recognized by the World Health Organization (WHO) as a Class II moderately hazardous herbicide, and its accidental or intentional ingestion can lead to multiorgan dysfunction and death. Overlap in presenting symptoms to other chemical poisonings makes it crucial to have a high clinical suspicion for this poisoning diagnosis. Herein, we present a case of an intentional overdose of 2,4-D and highlight the clinical presentation and treatment measures used.

## Introduction

Ethyl ester, chemically known as 2,4-dichlorophenoxyacetic acid (2,4-D), is a common herbicide against broadleaf weeds [1]. Human exposure can occur through direct contact, inhalation, or ingestion. Systemic toxicity with this agent is often associated with suicide attempts, seen chiefly in agricultural communities due to the use of this agent as an herbicide. Acute poisoning leads to myotoxicity, neurotoxicity, nephrotoxicity, cardiotoxicity, and even toxic hepatitis [1]. Initial symptoms mimic those caused by anticholinergics and sedative drugs, necessitating thorough history-taking and identification of the herbicide for accurate diagnosis and prompt management [2]. A peculiar odor can sometimes be noted in the breath of the affected individuals. Severe toxicity is characterized by pronounced rhabdomyolysis, metabolic acidosis, refractory hypotension, renal and respiratory failure, and coma [3]. Unfortunately, no specific antidote exists, so primary treatment focuses on supportive measures. Timely intervention with forced alkaline diuresis and hemodialysis may improve the prognosis [1,4].

## Case presentation

This report discusses a 52-year-old female individual admitted to the hospital following suicidal ingestion of an unknown quantity of "Sackweed-38," which contains 38% ethyl ester (2,4-D). The patient initially received medical treatment at a nearby hospital, where she had a few episodes of vomiting and diarrhea and developed an altered sensorium, which progressively worsened. She required intubation for airway protection, gastric lavage was performed, and she received intravenous fluids and needed initiation of vasopressors for management of hypotension.

On presentation to our emergency department, after approximately six hours of poison ingestion, she had a Glasgow Coma Score (GCS) of E1VTM1 and had pinpoint and nonreactive bilateral pupils. Vital signs on presentation included a blood pressure of 108/77 mmHg on norepinephrine and vasopressin support, a heart rate of 112/min, respiratory rate of 18/min, temperature of 98.6°F, and fingerstick glucose of 310 mg/dL. No salivation, lacrimation, fasciculations, or lung crepitations were observed on further examination.

**Figure 1:**
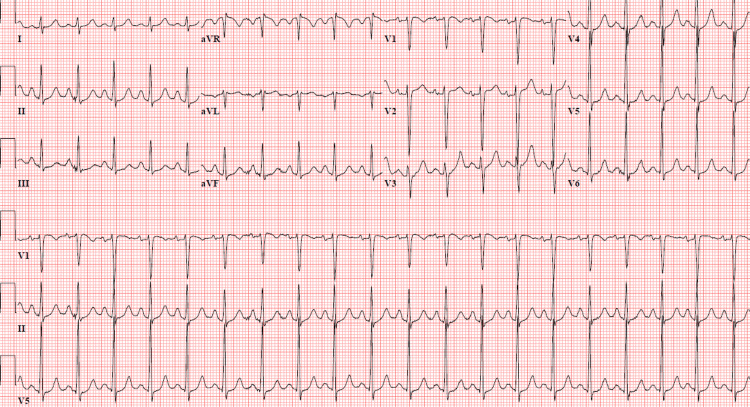
Electrocardiogram showing sinus tachycardia and non-specific T wave changes.

An electrocardiogram showed sinus tachycardia (Figure 1). Metabolic acidosis was seen on an arterial blood gas analysis. Other laboratory investigations are mentioned in Table 1. Blood levels of 2,4-D could not be measured due to the non-availability of this test at our facility. Emergent endoscopy revealed severe esophagitis with blood clots in the stomach. Due to the lack of a specific antidote, she was managed with supportive measures. She continued to have refractory hypotension throughout her hospitalization, necessitating continuous dual vasopressor support. She underwent alkaline diuresis with injectable sodium bicarbonate and maintained a urine output of 200 ml/hr. Other treatment measures included initiation of pantoprazole infusion and potassium supplementation for hypokalemia management. On the second day of hospital admission, her GCS slightly improved at E3VTM3, and her pupils became reactive to light. Neuromuscular symptoms of twitching, fasciculations, and hypotonia were noted, and an electroencephalogram ruled out seizure activity. She had fever spikes, for which empiric broad-spectrum antibiotics were added, and tracheal secretions were tested. The results showed the growth of multidrug-resistant Klebsiella pneumoniae and budding yeast cells. She received polymyxin B, ceftriaxone-sulbactam, and fluconazole based on the culture sensitivity results. Despite these measures, her oxygen requirements continued to increase, accompanied by worsening renal failure and metabolic acidosis. Electrolyte imbalance of hypernatremia persisted, and she became hyperkalemic due to deteriorating renal function. Unfortunately, she had a cardiac arrest and passed away on the sixth day of hospitalization.

**Table 1:** Laboratory investigations. BUN: Blood urea nitrogen; CPK: Creatine kinase; CRP: C-reactive protein; RBC: Red blood cell count; HPF: High-power field.

Labs	Reference range	Day:1	Day:3	Day: 5
Hemoglobin	12-15 gm/dL	11.2	8.9	7.4
White blood cell count (WBC)	4,000-11,000/µL	35	25.2	19.4
Platelet count	150,000-410,000/µL	302	206	150
BUN	7.9-20 mg/dL	16	30	65
Creatinine	0.6-1.1 mg/dL	1.08	1.67	3.31
Sodium	136-146 mmol/L	140	158	154
Potassium	3.5-5.1 mmol/L	2.59	3.33	5.51
Calcium	8.8-10.6 mg/dL	6.51	7.36	6.23
Bicarbonate	21-31 mmol/L	15.8	28.7	27.6
Lactate	0.5-1.5 mmol/L	5.1		
Total bilirubin	0.3-1.2 mg/dL	0.17		
Aspartate transaminase	<35 U/L	35		
Alanine transaminase	<35 U/L	22		
Alkaline phosphatase	30-120 U/L	69		
Albumin	3.5-5.0 g/dL	3.8		
CPK	10-120 µg/L	242	1027	
CRP	1-3 mg/L			441.73
Urine pH	4.5-8		6	
Urine specific gravity	1.005-1.030		1.010	
Urine color			Brownish-yellow	
Urine leukocyte esterase	Negative		Trace	
Urine nitrite	Negative		Negative	
Urine blood	Negative/Trace		Negative	
Urine bilirubin	Negative		Negative	
Urine RBC	0-3/HPF		0-3/HPF	
Urine WBC	0-5/HPF		15-20/HPF	
Urine microscopy			Muddy brown casts	
Urine protein	Negative, 10, 20		50	
Urine squamous epithelial cells	None/occasional		6-10/HPF	

## Discussion

2,4-D belongs to the chlorophenoxy group of herbicides. It is popularly used in parks, lawns, and fields of cereal crops to combat broadleaf weeds [1]. This odorless, colorless, or white crystalline powder is available in various concentrations ranging from 0.12% to 96.9%, and various chemical forms are available, including salts, esters, and an acid form. During the Vietnam War, it was one of the components of the herbicide used in chemical warfare, known as Agent Orange [3,5]. Humans can be exposed through direct eye or skin contact, inhalation, and even ingestion by spray, aerosol, and liquid forms of the herbicide [4,6]. Poisoning with this compound is rare but, if present, is associated with a high mortality rate of around 99.17% [7].

2,4-D toxin is suspected to induce dose-dependent mitochondrial and cell membrane damage, disrupting the acetyl coenzyme A metabolism, uncoupling of oxidative phosphorylation, and DNA damage by generating free radicals [5,8]. Disruption of cellular tubulin microtubule cytoskeleton network and p53-mediated mitochondrial apoptosis has also been observed in lung cells [6]. Neuromuscular toxicity occurs due to the inhibition of voltage-gated chloride channels in skeletal muscles [3]. Characteristic neurotoxic effects of 2,4-D arise from changes in the blood-brain barrier, causing structural and functional central nervous system degeneration [3]. Lung toxicity can result from the direct induction of cell death through microtubule network damage within lung cells [6]. A distinct feature of 2,4-D is muscle toxicity manifesting as rhabdomyolysis, triggering an acute increase in serum creatine kinase, metabolic acidosis, and ultimately precipitating renal failure [8]. 2,4-D also affects the nervous system, resulting in altered sensorium, coma, ataxia, and, less commonly, convulsions, fasciculations, and paralysis. Myopathic symptoms, including limb weakness, muscle spasms, and myotonia, are frequently seen [8].

Occupational exposure to 2,4-D spray can cause skin and eye irritations, itching, coughing, and breathing difficulties [6]. Patients who have acutely ingested this poison exhibit gastrointestinal symptoms such as vomiting, abdominal pain, diarrhea, and, in some cases, gastrointestinal bleeding due to corrosive chemical injury [1,8]. Examination findings can include hypotension and tachycardia from volume depletion due to gastrointestinal losses and the direct myocardial effect of 2,4-D [3]. Neurologically, patients may have hypo/hyperreflexia, ataxia, and fasciculations. Central nervous system depression and toxin-induced respiratory muscle weakness result in hypoventilation [8].

Laboratory investigations often reveal metabolic acidosis, derangements of renal function tests, and electrolyte imbalances. Elevated levels of creatine phosphokinase, lactate dehydrogenase, and sometimes abnormal liver function tests are observed. Additional signs of liver necrosis and fatty changes are also seen in some cases [9]. Cerebral edema may be seen in brain imaging, while myocardial injury causes generalized T wave changes in electrocardiograms [4]. The precise toxic and lethal levels of 2,4-D in human blood and tissues remain unclear. However, gas-liquid chromatography with electron capture can measure blood 2,4-D levels, in which lethal limits usually range from 447-826 mg/L [1].

Anticholinesterase poisons like organophosphates, carbamates, sedative drugs, or alcohol have mimicked initial symptoms of gastrointestinal distress, respiratory depression, and altered sensorium, increasing the risk of misdiagnosis [1,3]. However, key differentiating features of 2,4-D toxicity include the presence of rhabdomyolysis and signs of neuromuscular toxicity [3,5]. Acute kidney injury, significant rhabdomyolysis, and ventricular fibrillation become common causes of death in these rare poisonings [3,9].

Due to the nonavailability of a specific antidote, the mainstay of management includes immediate resuscitative and supportive measures [3]. Pre-hospital treatment of 2,4-D contact exposure should include removing contaminated clothes and cleaning the skin with alkaline, soapy water. In cases of contact with eyes, thorough washing with alkaline water is recommended [7]. In hospitals, patient care measures encompass airway and ventilatory support, gastrointestinal decontamination using activated charcoal or sorbitol, and gastric lavage [3]. Treatment for cases with metabolic acidosis, rhabdomyolysis, electrolyte imbalances, and hypotension includes crystalloid fluids, vasopressors, and rapid correction of electrolyte imbalances [3]. Patients with neurological abnormalities, convulsions, or seizures can be treated with diazepam [7]. The primary mode of 2,4-D removal is renal excretion. Injectable sodium bicarbonate with fluids and diuretics is used to induce urine alkalinization, causing ionization of the phenoxy acid and thus reducing reabsorption from renal tubules [3,9]. A target urine flow rate of 4-6 ml/min should be maintained [4]. Hemodialysis is the preferred treatment for severe cases, especially those with neurological damage [4]. If available, hemoperfusion therapy, including hemoperfusion, hemofiltration, hemodialysis, and plasma exchange, can be used in 2,4-D poisoning with central nervous toxicity [7].

## Conclusions

Accurate identification of the ingested chemical is crucial for appropriately managing cases with an alleged history of agrochemical poisonings. In patients with suspected agrochemical poisoning, a high clinical suspicion for 2,4-D weedicide poisoning and other common chemicals such as organophosphates and carbamates is vital for timely diagnosis. Initial identification, along with rapid decontamination and supportive care, may significantly influence patient outcomes.
